# 4E-BP1 counteracts human mesenchymal stem cell senescence via maintaining mitochondrial homeostasis

**DOI:** 10.1093/procel/pwac037

**Published:** 2022-08-23

**Authors:** Yifang He, Qianzhao Ji, Zeming Wu, Yusheng Cai, Jian Yin, Yiyuan Zhang, Sheng Zhang, Xiaoqian Liu, Weiqi Zhang, Guang-Hui Liu, Si Wang, Moshi Song, Jing Qu

**Affiliations:** State Key Laboratory of Membrane Biology, Institute of Zoology, Chinese Academy of Sciences, Beijing 100101, China; University of Chinese Academy of Sciences, Beijing 100049, China; State Key Laboratory of Membrane Biology, Institute of Zoology, Chinese Academy of Sciences, Beijing 100101, China; University of Chinese Academy of Sciences, Beijing 100049, China; State Key Laboratory of Membrane Biology, Institute of Zoology, Chinese Academy of Sciences, Beijing 100101, China; Beijing Institute for Stem Cell and Regenerative Medicine, Beijing 100101, China; Institute for Stem Cell and Regeneration, Chinese Academy of Sciences, Beijing 100101, China; State Key Laboratory of Membrane Biology, Institute of Zoology, Chinese Academy of Sciences, Beijing 100101, China; Beijing Institute for Stem Cell and Regenerative Medicine, Beijing 100101, China; Institute for Stem Cell and Regeneration, Chinese Academy of Sciences, Beijing 100101, China; State Key Laboratory of Membrane Biology, Institute of Zoology, Chinese Academy of Sciences, Beijing 100101, China; University of Chinese Academy of Sciences, Beijing 100049, China; Beijing Institute for Stem Cell and Regenerative Medicine, Beijing 100101, China; National Laboratory of Biomacromolecules, CAS Center for Excellence in Biomacromolecules, Institute of Biophysics, Chinese Academy of Sciences, Beijing 100101, China; University of Chinese Academy of Sciences, Beijing 100049, China; State Key Laboratory of Brain and Cognitive Science, CAS Center for Excellence in Brain Science and Intelligence Technology, Institute of Brain-Intelligence Technology (Shanghai), Institute of Biophysics, Chinese Academy of Sciences, Beijing 100101, China; State Key Laboratory of Stem Cell and Reproductive Biology, Institute of Zoology, Chinese Academy of Sciences, Beijing 100101, China; Beijing Institute for Stem Cell and Regenerative Medicine, Beijing 100101, China; Institute for Stem Cell and Regeneration, Chinese Academy of Sciences, Beijing 100101, China; University of Chinese Academy of Sciences, Beijing 100049, China; CAS Key Laboratory of Genomic and Precision Medicine, Beijing Institute of Genomics, Chinese Academy of Sciences, Beijing 100101, China; China National Center for Bioinformation, Beijing 100101, China; Aging Translational Medicine Center, International Center for Aging and Cancer, Beijing Municipal Geriatric Medical Research Center, Xuanwu Hospital, Capital Medical University, Beijing 100053, China; The Fifth People’s Hospital of Chongqing, Chongqing 400062, China; Institute for Stem Cell and Regeneration, Chinese Academy of Sciences, Beijing 100101, China; State Key Laboratory of Membrane Biology, Institute of Zoology, Chinese Academy of Sciences, Beijing 100101, China; Beijing Institute for Stem Cell and Regenerative Medicine, Beijing 100101, China; University of Chinese Academy of Sciences, Beijing 100049, China; Advanced Innovation Center for Human Brain Protection, and National Clinical Research Center for Geriatric Disorders, Xuanwu Hospital Capital Medical University, Beijing 100053, China; Institute for Stem Cell and Regeneration, Chinese Academy of Sciences, Beijing 100101, China; Advanced Innovation Center for Human Brain Protection, and National Clinical Research Center for Geriatric Disorders, Xuanwu Hospital Capital Medical University, Beijing 100053, China; Aging Translational Medicine Center, International Center for Aging and Cancer, Beijing Municipal Geriatric Medical Research Center, Xuanwu Hospital, Capital Medical University, Beijing 100053, China; The Fifth People’s Hospital of Chongqing, Chongqing 400062, China; State Key Laboratory of Membrane Biology, Institute of Zoology, Chinese Academy of Sciences, Beijing 100101, China; Beijing Institute for Stem Cell and Regenerative Medicine, Beijing 100101, China; University of Chinese Academy of Sciences, Beijing 100049, China; The Fifth People’s Hospital of Chongqing, Chongqing 400062, China; Institute for Stem Cell and Regeneration, Chinese Academy of Sciences, Beijing 100101, China; State Key Laboratory of Stem Cell and Reproductive Biology, Institute of Zoology, Chinese Academy of Sciences, Beijing 100101, China; Beijing Institute for Stem Cell and Regenerative Medicine, Beijing 100101, China; University of Chinese Academy of Sciences, Beijing 100049, China; Institute for Stem Cell and Regeneration, Chinese Academy of Sciences, Beijing 100101, China

**Keywords:** 4E-BP1, mitochondria, aging

## Abstract

Although the mTOR-4E-BP1 signaling pathway is implicated in aging and aging-related disorders, the role of 4E-BP1 in regulating human stem cell homeostasis remains largely unknown. Here, we report that the expression of 4E-BP1 decreases along with the senescence of human mesenchymal stem cells (hMSCs). Genetic inactivation of 4E-BP1 in hMSCs compromises mitochondrial respiration, increases mitochondrial reactive oxygen species (ROS) production, and accelerates cellular senescence. Mechanistically, the absence of 4E-BP1 destabilizes proteins in mitochondrial respiration complexes, especially several key subunits of complex III including UQCRC2. Ectopic expression of 4E-BP1 attenuates mitochondrial abnormalities and alleviates cellular senescence in 4E-BP1-deficient hMSCs as well as in physiologically aged hMSCs. These f indings together demonstrate that 4E-BP1 functions as a geroprotector to mitigate human stem cell senescence and maintain mitochondrial homeostasis, particularly for the mitochondrial respiration complex III, thus providing a new potential target to counteract human stem cell senescence.

## Introduction

Aging is a progressively degenerative process accompanied by stem cell attrition, thus leading to organ dysfunction (Oh *et al.*, 2014; Goodell and Rando, 2015; Neves *et al.*, 2017; Leng and Pawelec, 2022; Yang *et al.*, 2022). Stem cells maintain self-renewal and differentiate into multiple types of functional cells, playing a critical role in the regulation of tissue homeostasis and regeneration (Schultz and Sinclair, 2016; Ren *et al.*, 2017; Ermolaeva *et al.*, 2018). The dysregulation of tissue stem cells has been implicated in multiple age-associated disorders (Boyette and Tuan, 2014; Neves *et al.*, 2017). Mesenchymal stem cells (MSCs) are a type of adult stem cells resident in multiple tissues and can differentiate into various types of cells such as osteoblasts, chondrocytes, and adipocytes (Fehrer and Lepperdinger, 2005; Bianco, 2014; Zhao and Chen, 2022). The senescence of MSCs is associated with the aging of multiple organs, yet the molecular mechanisms for the regulation of MSC senescence remain elusive.

Eukaryotic translation initiation factor 4E-binding protein 1 (4E-BP1), encoded by *EIF4EBP1* gene, is a key substrate and downstream effector of the mechanistic target of rapamycin complex 1 (mTORC1), implicated in a variety of physiological and pathological processes including aging and cancer (Schalm *et al.*, 2003; Weichhart, 2018). As a master regulator of mRNA translation, the active form of 4E-BP1 binds to eukaryotic translation initiation factor 4E (eIF4E), and thus inhibits the cap-dependent translation initiation (Choo *et al.*, 2008; Musa *et al.*, 2016; Qin *et al.*, 2016). It has been reported that the *Drosophila* homolog of 4E-BP1 is upregulated upon dietary restriction (DR) in *Drosophila*, mediating the beneficial effects of DR by the translational regulation of nuclear-encoded mitochondrial genes (Zid *et al.*, 2009). However, it is unclear whether 4E-BP1 plays a critical role in regulating the homeostasis or senescence of human stem cells.

In this study, we find that the expression of 4E-BP1 is decreased in replicatively and physiologically senescent human MSCs (hMSCs) and that 4E-BP1 deficiency accelerates hMSC senescence. Specifically, the loss of 4E-BP1 increases mitochondrial reactive oxygen species (ROS) levels while decreases the capability of mitochondrial respiration. Re-introduction of 4E-BP1 upregulates the expression of mitochondrial oxidative phosphorylation complex III core component UQCRC2, thus partially restoring the mitochondrial homeostasis and rejuvenating senescent hMSCs. Altogether, our study identifies a novel role of 4E-BP1 in maintaining mitochondrial homeostasis and alleviating cellular senescence in hMSCs.

## Results

### Depletion of 4E-BP1 accelerates hMSC senescence

**Figure 1.**
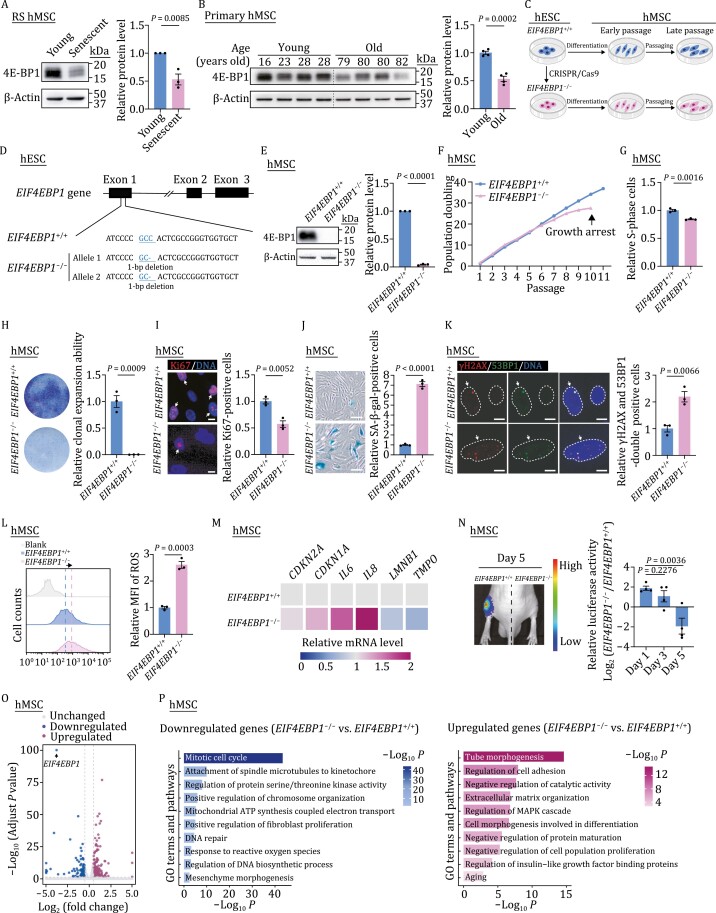
4E-BP1-deficient hMSCs exhibit phenotypes of accelerated senescence. (A) Western blot analysis of 4E-BP1 in young (P4) and senescent (P16) hMSCs. β-Actin was used as a loading control. Statistical data are presented as means ± SEM. *n* = 3 independent experiments. Two-tailed unpaired *t*-test. (B) Western blot analysis of 4E-BP1 in primary hMSCs derived from young and old individuals. β-Actin was used as a loading control. Statistical data are presented as means ± SEM. *n* = 4 biological replicates. Two-tailed unpaired *t*-test. (C) Schematic diagram for the generation of *EIF4EBP1*^+/+^ and *EIF4EBP1*^−/−^ hESCs and hMSCs. (D) Schematic of the deletion of 4E-BP1 via CRISPR/Cas9-mediated nonhomologous end-joining (NHEJ). Sequencing results showed a 1-bp (C/G) deletion introduced in exon 1 by genome editing. (E) Western blot analysis of 4E-BP1 in *EIF4EBP1*^+/+^ and *EIF4EBP1*^−/−^ hMSCs (P4). β-Actin was used as a loading control. Statistical data are presented as means ± SEM. *n* = 3 independent experiments. Two-tailed unpaired *t*-test. (F) Growth curve analysis of *EIF4EBP1*^+/+^ and *EIF4EBP1*^−/−^ hMSCs. Data are representative of two independent experiments. (G) Cell cycle analysis of *EIF4EBP1*^+/+^ and *EIF4EBP1*^−/−^ hMSCs (P9). Statistical data are presented as means ± SEM. *n* = 3 biological replicates. Two-tailed unpaired *t*-test. (H) Clonal expansion assay in *EIF4EBP1*^+/+^ and *EIF4EBP1*^−/−^ hMSCs (P9). Statistical data are presented as the means ± SEM. *n* = 3 biological replicates. Two-tailed unpaired *t*-test. (I) Immunofluorescence analysis of Ki67 in *EIF4EBP1*^+/+^ and *EIF4EBP1*^−/−^ hMSCs (P9). Arrows indicate Ki67-positive cells. Scale bars, 10 μm. Statistical data are presented as means ± SEM. *n* = 3 biological replicates. Two-tailed unpaired *t*-test. (J) SA-β-gal staining of *EIF4EBP1*^+/+^ and *EIF4EBP1*^−/−^ hMSCs (P9). Scale bars, 100 μm. Statistical data showing the relative SA-β-gal-positive cells are presented as means ± SEM to the right. *n* = 3 biological replicates. Two-tailed unpaired *t*-test. (K) Immunofluorescence analysis of γH2AX and 53BP1 in *EIF4EBP1*^+/+^ and *EIF4EBP1*^−/−^ hMSCs (P9). Arrows indicate γH2AX and 53BP1 double-positive cells. Dashed lines represent nuclear boundaries of hMSCs. Scale bars, 10 μm. Statistical data are presented as means ± SEM. *n* = 3 biological replicates. Two-tailed unpaired *t*-test. (L) Flow cytometric analysis of ROS in *EIF4EBP1*^+/+^ and *EIF4EBP1*^−/−^ hMSCs (P9). Dashed lines indicate the position of mean fluorescence intensity (MFI) in *EIF4EBP1*^+/+^ and *EIF4EBP1*^−/−^ hMSCs. Statistical data (right) showing the relative fold of MFI are presented as means ± SEM. *n* = 3 biological replicates. Two-tailed unpaired *t*-test. (M) Heatmap showing the relative mRNA levels of *CDKN2A*, *CDKN1A*, *IL6*, *IL8*, *LMNB1* and *TMPO* in *EIF4EBP1*^−/−^ hMSCs compared with *EIF4EBP1*^+/+^ hMSCs (P9). Data are representative of two independent experiments. (N) Analysis of luciferase activities in tibialis anterior (TA) muscles of nude mice implanted with *EIF4EBP1*^+/+^ (left) and *EIF4EBP1*^−/−^ (right) hMSCs (P8) transduced with lentiviruses expressing luciferase. Data are obtained at day 1, day 3 and day 5 after implantation, and presented as means ± SEM. *n* = 4 mice. Two-tailed unpaired *t*-test. (O) Volcano plot showing the differentially expressed genes (DEGs) in *EIF4EBP1*^−/−^ hMSCs compared with *EIF4EBP1*^+/+^ hMSCs (P8). (P) Gene Ontology (GO) enrichment analysis of significantly downregulated (left) or upregulated (right) genes in *EIF4EBP1*^−/−^ hMSCs compared with *EIF4EBP1*^+/+^ hMSCs (P8).

To examine whether the expression of 4E-BP1 was altered during human stem cell senescence, we detected the level of 4E-BP1 in young and senescent hMSCs by western blotting and found that it was decreased in replicatively senescent hMSCs (Fig. 1A). Similarly, the expression of 4E-BP1 was lower in primary hMSCs isolated from aged individuals than that from young individuals (Fig. 1B). To further explore the role of 4E-BP1 in hMSC senescence, we generated 4E-BP1-deficient (*EIF4EBP1*^−/−^) human embryonic stem cells (hESCs) via CRISPR/Cas9-based gene-editing technique and then differentiated them into *EIF4EBP1*^−/−^ hMSCs (Fig. 1C and 1D). The deletion of 4E-BP1 in hESCs was confirmed by western blotting (Fig. S1A). The genomic integrity in *EIF4EBP1*^−/−^ hESCs was well maintained as revealed by karyotyping and genome-wide copy number variation (CNV) analyses (Fig. S1B and S1C). No off-target cleavage was detected at the predicted genomic locus with relatively high off-target scores (Fig. S1D). In addition, *EIF4EBP1*^−/−^ hESCs retained the expression of pluripotency markers, including OCT4, SOX2, and NANOG (Fig. S1E and S1F). Furthermore, the deficiency of 4E-BP1 did not adversely affect the percentage of S-phase cells in hESCs (Fig. S1G). These results demonstrate that 4E-BP1 decreases in senescent hMSCs but that it is potentially dispensable for the maintenance of self-renewal and pluripotency of hESCs.

Then, we differentiated wild-type (WT, *EIF4EBP1*^+/+^) and *EIF4EBP1*^−/−^ hESCs into hMSCs to evaluate the effect of 4E-BP1 deficiency in hMSCs (Fig. 1C). The deletion of 4E-BP1 in hMSCs was verified by western blotting (Fig. 1E). Flow cytometry analysis showed that both *EIF4EBP1*^+/+^ and *EIF4EBP1*^−/−^ hMSCs expressed canonical hMSC markers, such as CD73, CD90, CD105, CD166, CD29, CD44, CD13, and HLA, but not non-hMSC markers, including CD34, CD43, CD45, CD14, CD19, CD164, and PDPN (Fig. S2A and S2B). Similar to *EIF4EBP1*^+/+^ hMSCs, *EIF4EBP1*^−/−^ hMSCs at early passage could differentiate into osteoblasts and adipocytes (Fig. S2C and S2D) and maintained normal morphology and proliferation potential (Fig. S2E–H). However, with passaging, the cellular proliferative ability in *EIF4EBP1*^−/−^ hMSCs was reduced compared with that in *EIF4EBP1*^+/+^ hMSCs, as revealed by early-onset growth arrest, decreased percentage of S-phase cells, compromised clonal expansion capacity, and diminished Ki67-positive cells (Fig. 1F–I). Moreover, *EIF4EBP1*^−/−^ hMSCs exhibited accelerated cellular senescence, as manifested by increased SA-β-gal-positive cells and DNA damage, and accumulated ROS (Fig. 1J–L). The quantitative reverse-transcription PCR (qRT-PCR) analysis showed that the expression of senescence markers, such as *CDKN2A* (*P16*) and *CDKN1A* (*P21*), and senescence-associated secretory phenotype (SASP) factors, including *IL6* and *IL8*, also increased in 4E-BP1-deficient hMSCs (Fig. 1M). Meanwhile, the levels of nuclear lamina protein Lamin B1 (*LMNB1*) and lamina-associated protein LAP2 (*TMPO*) were diminished in *EIF4EBP1*^−/−^ hMSCs relative to *EIF4EBP1*^+/+^ hMSCs (Fig. 1M), consistent with previous findings in senescent hMSCs (Bi *et al.*, 2020; Diao *et al.*, 2021; Sun *et al.*, 2022). Furthermore, we observed an accelerated decay of *EIF4EBP1*^−/−^ hMSCs *in vivo* when implanted into the tibialis anterior (TA) muscles of nude mice, suggesting impaired *in vivo* retention ability of *EIF4EBP1*^−/−^ hMSCs (Fig. 1N). Next, we performed RNA-sequencing (RNA-seq) analysis of *EIF4EBP1*^+/+^ and *EIF4EBP1*^−/−^ hMSCs to dissect the potential mechanisms by which 4E-BP1 regulates hMSC senescence (Figs. 1O and S2I; Table S1). Consistent with the senescence phenotypes in *EIF4EBP1*^−/−^ hMSCs, Gene Ontology (GO) analysis revealed that genes associated with mitotic cell cycle, DNA repair, and response to ROS were downregulated in *EIF4EBP1*^−/−^ hMSCs (Fig. 1O and 1P). By contrast, genes associated with regulation of cell adhesion, negative regulation of catalytic activity, negative regulation of protein maturation, and aging were increased in *EIF4EBP1*^−/−^ hMSCs (Fig. 1O and 1P). Collectively, these results indicate that 4E-BP1 deficiency accelerates hMSC senescence.

### 4E-BP1 deficiency compromises mitochondrial respiration

**Figure 2.**
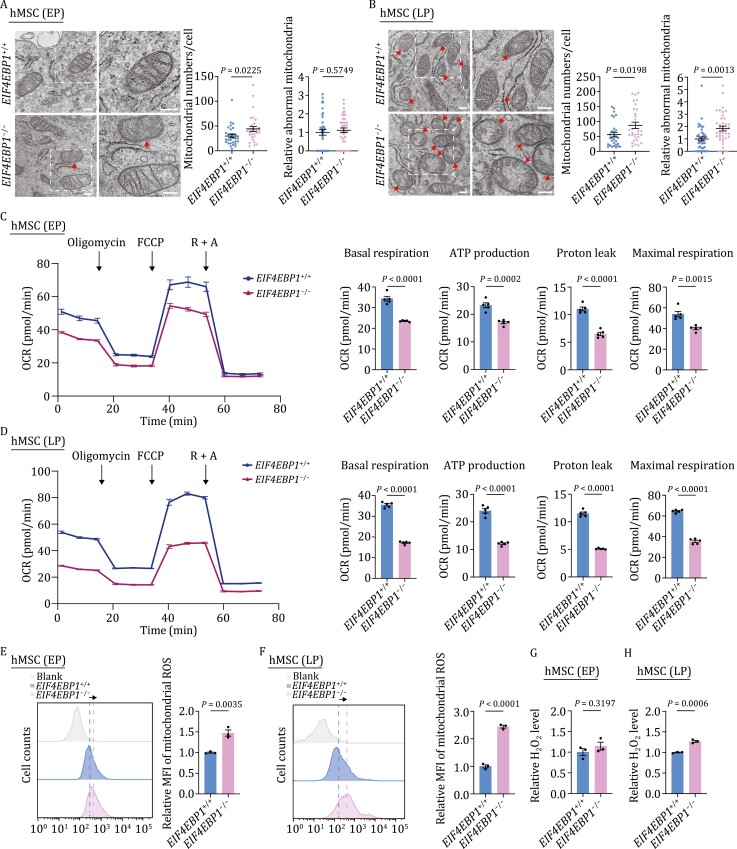
Deficiency of 4E-BP1 impairs mitochondrial respiration and induces mitochondrial ROS. (A, B) Transmission electron microscopy analysis of mitochondrial numbers per cell and abnormal mitochondria rates of *EIF4EBP1*^+/+^ and *EIF4EBP1*^−/−^ hMSCs at early passage (EP, P4) (A) and late passage (LP, P9) (B). Representative electron micrograph on the left shows the magnified mitochondria-rich region in a single cell. Representative image on the right shows the zoomed-in area of the white dashed box. Arrows indicate abnormal mitochondrias. Scale bars, 200 nm. Statistical data showing mitochondrial numbers per intact cell are presented as means ± SEM. *n* = 30 cells. Two-tailed unpaired *t*-test. Statistical data showing the relative percentages of abnormal mitochondria are presented as means ± SEM. 40 images were calculated to determine the relative percentage of abnormal mitochondria in each sample. Two-tailed unpaired *t*-test. (C, D) Detection of the oxygen consumption rates (OCR) in *EIF4EBP1*^+/+^ and *EIF4EBP1*^−/−^ hMSCs at EP (P4) (C) and LP (P9) (D) in response to indicated mitochondrial modulators (Oligomycin, FCCP, Rotenone and Antimycin A [R+A]). Basal respiration, ATP production, proton leak, and maximal respiration were calculated by the OCR values of *EIF4EBP1*^+/+^ and *EIF4EBP1*^−/−^ hMSCs. Statistical data are presented as means ± SEM, *n* = 5 biological replicates. Two-tailed unpaired *t*-test. (E, F) Flow cytometric analysis of mitochondrial ROS with MitoSOX™ Red in *EIF4EBP1*^+/+^ and *EIF4EBP1*^−/−^ hMSCs at EP (P4) (E) and LP (P9) (F). Dashed lines indicate the position of MFI in *EIF4EBP1*^+/+^ and *EIF4EBP1*^−/−^ hMSCs. Statistical data are presented as means ± SEM, *n* = 3 biological replicates. Two-tailed unpaired *t*-test. (G, H) Detection of H_2_O_2_ level with Amplex Red in *EIF4EBP1*^+/+^ and *EIF4EBP1*^−/−^ hMSCs at EP (P4) (G) and LP (P9) (H). Statistical data are presented as means ± SEM. *n* = 3 biological replicates. Two-tailed unpaired *t*-test.

Mitochondrial dysfunction has been implicated in human stem cell senescence (Wang *et al.*, 2020; Diao *et al.*, 2021). Considering a previous report that 4E-BP1 regulates the mitochondrial oxidative phosphorylation (OXPHOS) activity (Zid *et al.*, 2009), we hypothesized that hMSC senescence induced by 4E-BP1 depletion may be at least partially due to the impairment of mitochondrial fitness. To this end, we performed the electron microscopy analysis and found increased mitochondrial abundance at both early and late passages and more abnormalities at late passage in *EIF4EBP1*^−/−^ hMSCs compared with those in *EIF4EBP1*^+/+^ hMSCs (Fig. 2A and 2B). In addition, we found that 4E-BP1 depletion impaired mitochondrial respiration, as evidenced by decreased basal respiration, ATP production, proton leak, and maximal respiration (Fig. 2C and 2D). Consistent with a previous study suggesting that a dysfunctional electron transport chain may lead to ROS accumulation (Chen *et al.*, 2003; Kong *et al.*, 2022), we observed increased mitochondrial ROS and cellular H_2_O_2_ levels in *EIF4EBP1*^−/−^ hMSCs (Fig. 2E–H). Altogether, these findings indicate that 4E-BP1 plays an important role in the maintenance of mitochondrial fitness.

### Downregulation of key subunits of mitochondrial complex III in 4E-BP1-deficient hMSCs

**Figure 3.**
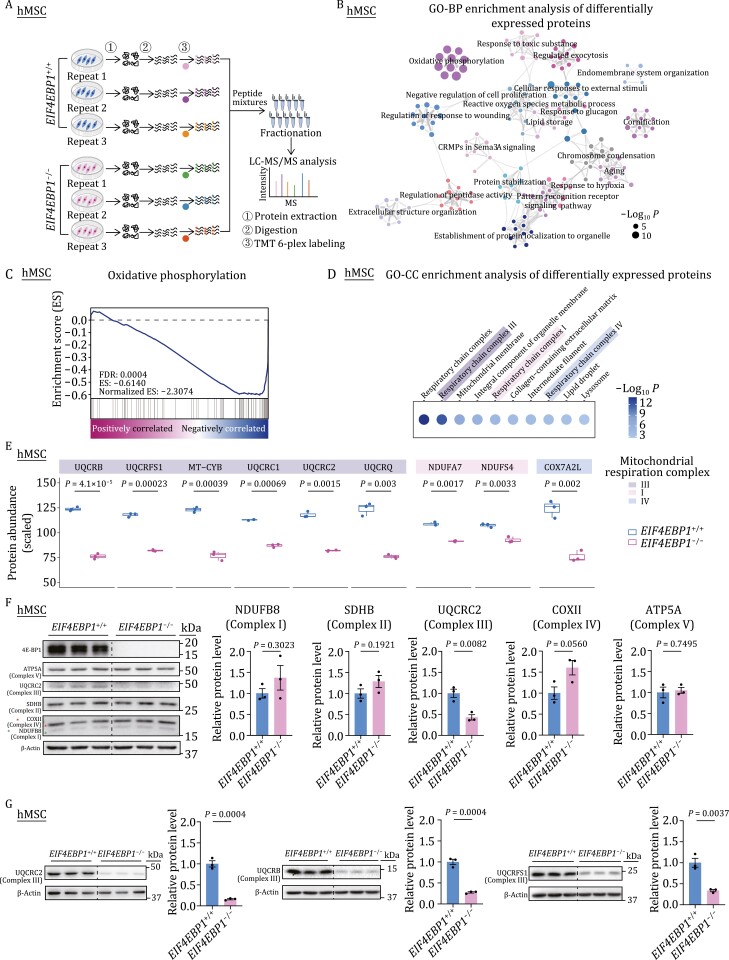
Depletion of 4E-BP1 in hMSCs reduces the levels of core components of the mitochondrial respiration complex III. (A) Schematic diagram of quantitative proteomic analysis. (B) Gene Ontology Biological Process (GO-BP) enrichment analysis of differentially expressed proteins (DEPs) between *EIF4EBP1*^+/+^ and *EIF4EBP1*^−/−^ hMSCs (P4). (C) GSEA of a statistically significant gene set, oxidative phosphorylation, in *EIF4EBP1*^−/−^ hMSCs compared with *EIF4EBP1*^+/+^ hMSCs (P4). (D) Gene Ontology Cellular Component (GO-CC) enrichment analysis of DEPs between *EIF4EBP1*^+/+^ and *EIF4EBP1*^−/−^ hMSCs (P4). (E) Boxplots showing the scaled protein abundance of DEPs of mitochondrial complexes I, III, and IV in *EIF4EBP1*^−/−^ hMSCs compared with *EIF4EBP1*^+/+^ hMSCs (P4). *n* = 3 biological replicates. Two-tailed unpaired *t*-test. (F) Western blot analysis of components of mitochondrial respiration complexes I, II, III, IV, V using a total OXPHOS human WB antibody cocktail (Abcam ab110411) in *EIF4EBP1*^+/+^ and *EIF4EBP1*^−/−^ hMSCs (P4). Statistical data are presented as means ± SEM. *n* = 3 biological replicates. Two-tailed unpaired *t*-test. (G) Western blot analysis of UQCRC2, UQCRB and UQCRFS1 using independent antibodies in *EIF4EBP1*^+/+^ and *EIF4EBP1*^−/−^ hMSCs (P4). Antibodies for UQCRC2 (Santa sc-390378), UQCRB (Abcam ab190360) and UQCRFS1 (Santa sc-271609) were utilized for targeting UQCRC2, UQCRB and UQCRFS1, respectively. Statistical data are presented as means ± SEM. *n* = 3 biological replicates. Two-tailed unpaired *t*-test.

To further unveil the molecular mechanism and key downstream targets underlying the mitochondrial dysregulation in 4E-BP1-deficient hMSCs, we performed quantitative proteomic analysis to identify differences in protein levels between *EIF4EBP1*^−/−^ hMSCs and their WT counterparts (Figs. 3A and S2J; Table S2). Intriguingly, functional enrichment analysis revealed that proteins related to OXPHOS were most affected upon 4E-BP1 depletion (Fig. 3B). Moreover, differentially expressed proteins in the oxidative phosphorylation pathway were mostly downregulated, especially those in respiratory chain complexes I, III, and IV (Fig. 3C and 3D). Specifically, among the OXPHOS proteins, the decreased ones were mainly found in the complex III, including UQCRB, UQCRFS1, MT-CYB, UQCRC1, UQCRC2, and UQCRQ (Fig. 3E), while only two components (NDUFA7 and NDUFS4) of complex I and one (COX7A2L) of complex IV were downregulated in hMSCs lacking 4E-BP1 (Fig. 3E). Western blot analysis further validated the diminishment of the complex III component, but not those from other complexes, in *EIF4EBP1*^−/−^ hMSCs (Fig. 3F), consistent with the observations of impaired mitochondrial respiration and increased ROS production in *EIF4EBP1*^−/−^ hMSCs. Particularly, multiple components of complex III, not only UQCRC2, but also UQCRB and UQCRFS1, were all downregulated, as revealed by western blot results (Fig. 3F and 3G). Taken together, these results demonstrate that the deficiency of 4E-BP1 decreases the expression of mitochondrial OXPHOS complex III subunits.

### Impaired mitochondrial respiration complex III contributes to cellular senescence

**Figure 4.**
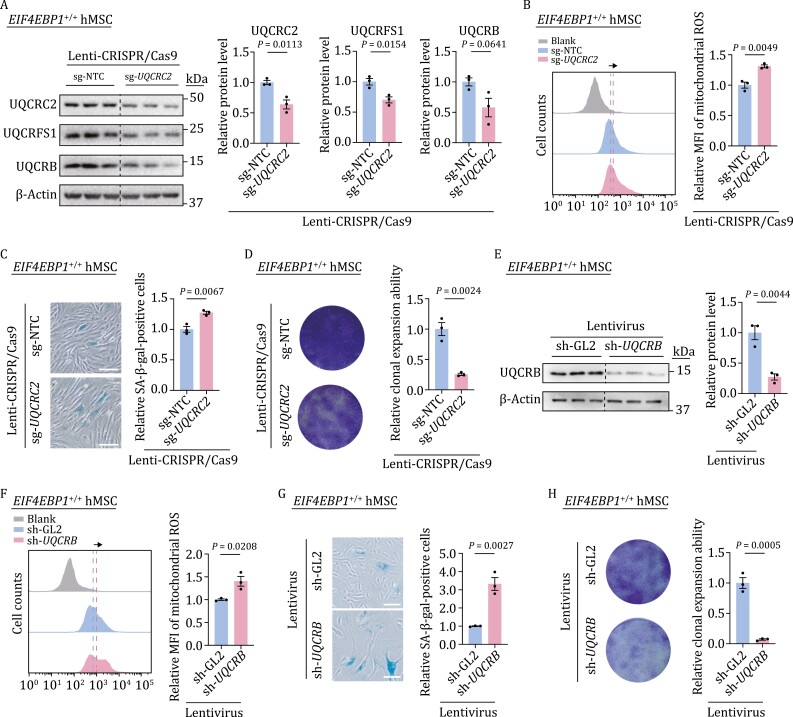
Deficiency of mitochondrial respiration complex III induces hMSC senescence. (A) Western blot analysis of UQCRC2, UQCRFS1, UQCRB in *EIF4EBP1*^+/+^ hMSCs (P4) transduced with lentiviruses expressing sg-NTC or sg-*UQCRC2*. Statistical data are presented as means ± SEM. *n* = 3 biological replicates. Two-tailed unpaired *t*-test. (B) Flow cytometric analysis of mitochondrial ROS in *EIF4EBP1*^+/+^ hMSCs (P4) transduced with lentiviruses expressing sg-NTC or sg-*UQCRC2*. Dashed lines indicate the position of MFI in sg-NTC or sg-*UQCRC2* expressing hMSCs. Statistical data are presented as means ± SEM. *n* = 3 biological replicates. Two-tailed unpaired *t*-test. (C) SA-β-gal staining of *EIF4EBP1*^+/+^ hMSCs (P4) transduced with lentiviruses expressing sg-NTC or sg-*UQCRC2*. Scale bars, 100 μm. Statistical data are presented as means ± SEM. *n* = 3 biological replicates. Two-tailed unpaired *t*-test. (D) Clonal expansion assay in *EIF4EBP1*^+/+^ hMSCs (P4) transduced with lentiviruses expressing sg-NTC or sg-*UQCRC2*. Statistical data are presented as means ± SEM. *n* = 3 biological replicates. Two-tailed unpaired *t*-test. (E) Western blot analysis of UQCRB in *EIF4EBP1*^+/+^ hMSCs (P4) transduced with lentiviruses expressing sh-GL2 or sh-*UQCRB*. Statistical data are presented as means ± SEM. *n* = 3 biological replicates. Two-tailed unpaired *t*-test. (F) Flow cytometric analysis of mitochondrial ROS in *EIF4EBP1*^+/+^ hMSCs (P4) transduced with lentiviruses expressing sh-GL2 or sh-*UQCRB*. Dashed lines indicate the position of MFI in sh-GL2 or sh-*UQCRB* expressing hMSCs. Statistical data are presented as means ± SEM. *n* = 3 biological replicates. Two-tailed unpaired *t*-test. (G) SA-β-gal staining of *EIF4EBP1*^+/+^ hMSCs (P4) transduced with lentiviruses expressing sh-GL2 or sh-*UQCRB*. Scale bars, 100 μm. Statistical data are presented as means ± SEM. *n* = 3 biological replicates. Two-tailed unpaired *t*-test. (H) Clonal expansion assay in *EIF4EBP1*^+/+^ hMSCs (P4) transduced with lentiviruses expressing sh-GL2 or sh-*UQCRB*. Statistical data are presented as means ± SEM. *n* = 3 biological replicates. Two-tailed unpaired *t*-test.

To further evaluate the role of the mitochondrial respiration complex III in regulating hMSC senescence, we knocked down UQCRC2, a core subunit of mitochondrial respiratory complex III, in *EIF4EBP1*^+/+^ hMSCs using lentiviral CRISPR/Cas9-mediated gene editing. Western blot analysis verified the decrease of UQCRC2, the accompanying downregulation of UQCRFS1, and a tendency of decreased UQCRB (Fig. 4A). Subsequently, we found that UQCRC2 deficiency resulted in dysregulated mitochondrial homeostasis in hMSCs, as evidenced by the accumulation of mitochondrial ROS (Fig. 4B). Furthermore, UQCRC2 deficiency induced hMSC senescence phenotypes, including increased SA-β-gal-positive cells and decreased colony formation ability (Fig. 4C and 4D). To further examine the potential effects of other components of the complex III downregulated in *EIF4EBP1*^−/−^ hMSCs, we knocked down UQCRB in *EIF4EBP1*^+/+^ hMSCs and observed increased mitochondrial ROS levels (Fig. 4E and 4F). Likewise, UQCRB deficiency also increased SA-β-gal-positive cells and decreased colony formation ability, suggesting accelerated senescence in hMSCs (Fig. 4G and 4H). These data indicate that the impairment of mitochondrial OXPHOS complex III core components disrupts the mitochondrial fitness and induces cellular senescence in hMSCs.

### 4E-BP1 stabilizes a core component of OXPHOS complex III, UQCRC2

**Figure 5.**
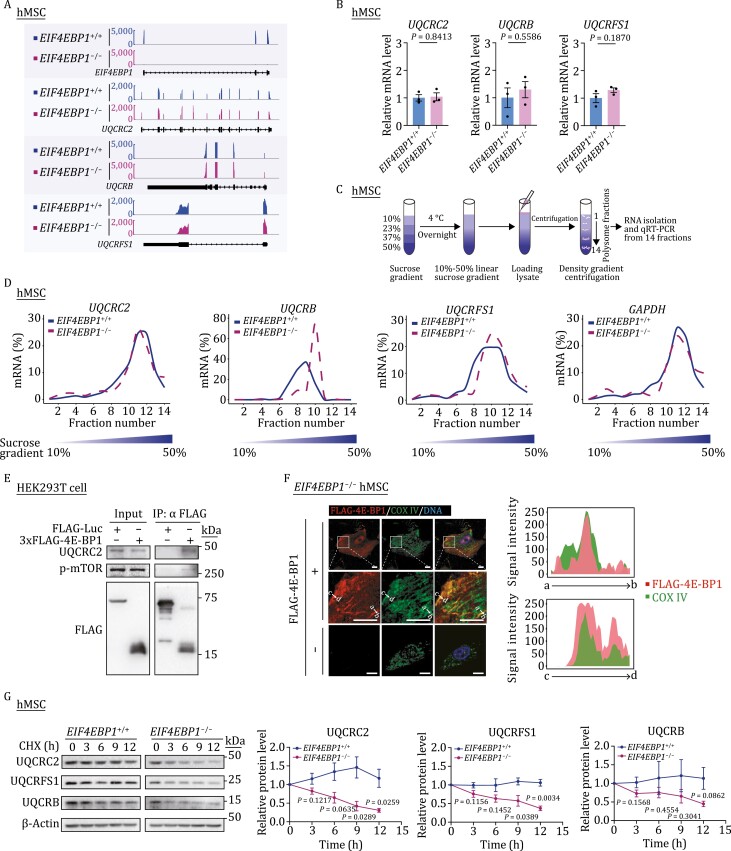
4E-BP1 stabilizes mitochondrial OXPHOS complex III core component UQCRC2. (A) Representative tracks showing the mRNA abundance of *EIF4EBP1*, *UQCRC2*, *UQCRB* and *UQCRFS1* in *EIF4EBP1*^+/+^ and *EIF4EBP1*^−/−^ hMSCs (P4). (B) mRNA levels of *UQCRC2*, *UQCRB* and *UQCRFS1* in *EIF4EBP1*^+/+^ and *EIF4EBP1*^−/−^ hMSCs (P4) analyzed by qRT-PCR. Statistical data are presented as means ± SEM. *n* = 3 biological replicates. Two-tailed unpaired *t*-test. (C) Schematic diagram of polysome profiling for translational analysis. (D) Density plots showing the distribution of *UQCRC2*, *UQCRB*, *UQCRFS1*, and *GAPDH* mRNAs over the sucrose gradient analyzed by qRT-PCR in hMSCs (P4). (E) Co-IP analysis of interactions between 3×FLAG-4E-BP1 and indicated proteins in HEK293T cells. (F) Immunofluorescence analysis of the mitochondrial inner membrane protein COX IV and FLAG-4E-BP1 in *EIF4EBP1*^−/−^ hMSCs transduced with or without lentiviruses expressing FLAG-4E-BP1. Scale bars, 10 μm. Fluorescence intensity plots (right) showing the distributions of FLAG-4E-BP1 and COX IV signals at the indicated regions (white arrows indicate a to b or c to d regions) in *EIF4EBP1*^−/−^ hMSCs. (G) Protein stability analysis of UQCRC2, UQCRFS1 and UQCRB in *EIF4EBP1*^+/+^ and *EIF4EBP1*^−/−^ hMSCs (P4). Protein levels of UQCRC2, UQCRFS1 and UQCRB at indicated time points in the presence of a protein synthesis inhibitor cycloheximide (CHX) were determined by western blot. In order to quantify in a suitable gray value, images of *EIF4EBP1*^+/+^ hMSCs in relatively shorter exposure time were obtained for quantification of UQCRC2, UQCRFS1 and UQCRB, while images of EIF4EBP1^−/−^ hMSCs in relatively longer exposure time were obtained for quantification of UQCRC2, UQCRFS1 and UQCRB. Statistical data are presented as means ± SEM. *n* = 3 independent experiments. Two-tailed unpaired *t*-test.

To understand how 4E-BP1 regulated the expression of OXPHOS complex III subunits, including UQCRC2, UQCRB, and UQCRFS1, we interrogated their changes at the transcriptional and translational levels. Transcriptionally, we detected no significant difference in the mRNA levels of UQCRC2, UQCRB, and UQCRFS1 between *EIF4EBP1*^+/+^ and *EIF4EBP1*^−/−^ hMSCs by RNA-seq and qRT-PCR analyses (Fig. 5A and 5B). At the translational level, we performed polysome profiling to measure the translational status of UQCRC2, UQCRB, and UQCRFS1 in *EIF4EBP1*^+/+^ and *EIF4EBP1*^−/−^ hMSCs (Fig. 5C and 5D). Although 4E-BP1 has been reported as a translation repressor in certain mRNAs with a special structure (Thoreen *et al.*, 2012), here we found that the translational efficiency of UQCRC2 was unaffected by 4E-BP1 deficiency in hMSCs (Fig. 5D). Conversely, increased translation of UQCRB and a trend of slightly increased translation of UQCRFS1 were observed upon 4E-BP1 ablation (Fig. 5D). These results could not explain reduced UQCRB and UQCRFS1 protein levels in 4E-BP1-deficient hMSCs. Given that 4E-BP1 has been reported to regulate the stability of p21 protein (Llanos *et al.*, 2016), we then examined whether it modulated the stability of OXPHOS complex III proteins in hMSCs. Indeed, we first identified a potential interaction between 4E-BP1 and UQCRC2 using co-immunoprecipitation (co-IP) assay (Fig. 5E). In addition, immunofluorescence staining revealed that a portion of 4E-BP1 signals colocalized with COX IV, a mitochondrial inner ­membrane protein also belonging to mitochondrial OXPHOS (Fig. 5F). Furthermore, we found accelerated degradation of UQCRC2 upon cycloheximide (CHX) treatment in *EIF4EBP1*^−/−^ hMSCs compared with that in *EIF4EBP1*^+/+^ hMSCs (Fig. 5G), implying a role of 4E-BP1 in the stabilization of UQCRC2. Moreover, we observed an accompanying decrease in the protein stability of UQCRFS1, as well as a decreasing trend in that of UQCRB, in *EIF4EBP1*^−/−^ hMSCs (Fig. 5G). Altogether, these results indicate that 4E-BP1 regulates mitochondrial homeostasis probably in part through the stabilization of mitochondrial OXPHOS complex III core components.

### Overexpression of 4E-BP1 rescues mitochondrial homeostasis and alleviates hMSC senescence

**Figure 6.**
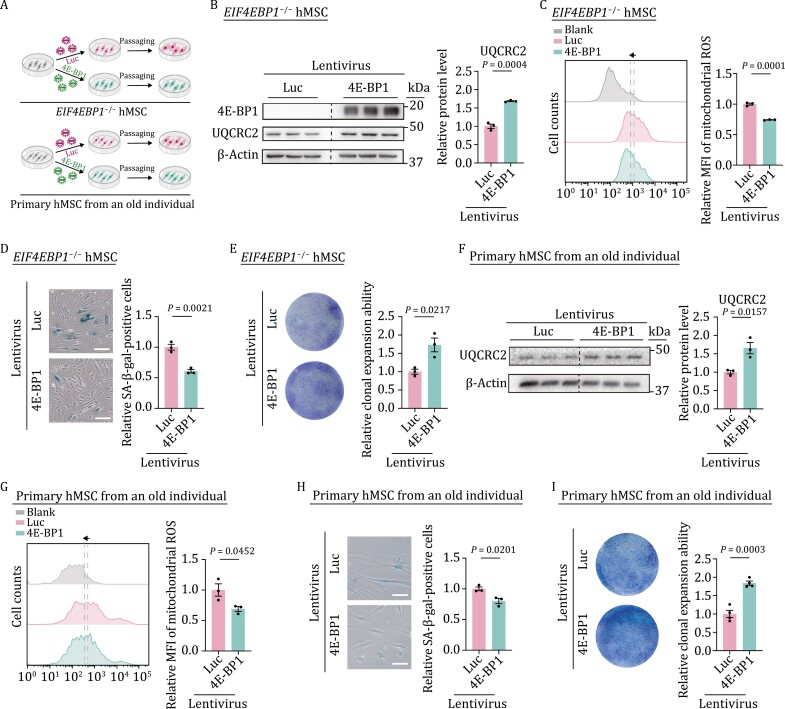
Overexpression of 4E-BP1 rescues mitochondrial dyshomeostasis and alleviates senescence. (A) Schematic diagram showing the ectopic expression of luciferase (Luc) or 4E-BP1 in *EIF4EBP1*^−/−^ hMSCs or primary hMSCs from an old individual. (B) Western blot analysis of UQCRC2 in *EIF4EBP1*^−/−^ hMSCs (P3) transduced with lentiviruses expressing Luc or 4E-BP1. Statistical data are presented as means ± SEM. *n* = 3 biological replicates. Two-tailed unpaired *t*-test. (C) Flow cytometric analysis of mitochondrial ROS in *EIF4EBP1*^−/−^ hMSCs (P3) transduced with lentiviruses expressing Luc or 4E-BP1. Dashed lines indicate the position of MFI in Luc or 4E-BP1 expressing hMSCs. Statistical data are presented as means ± SEM. *n* = 3 biological replicates. Two-tailed unpaired *t*-test. (D) SA-β-gal staining of *EIF4EBP1*^−/−^ hMSCs (P3) transduced with lentiviruses expressing Luc or 4E-BP1. Scale bars, 100 μm. Statistical data are presented as means ± SEM. *n* = 3 biological replicates. Two-tailed unpaired *t*-test. (E) Clonal expansion assay in *EIF4EBP1*^−/−^ hMSCs (P3) transduced with lentiviruses expressing Luc or 4E-BP1. Statistical data are presented as means ± SEM. *n* = 3 biological replicates. Two-tailed unpaired *t*-test. (F) Western blot analysis of UQCRC2 in primary hMSCs isolated from a 79-year-old individual transduced with lentiviruses expressing Luc or 4E-BP1. Statistical data are presented as means ± SEM. *n* = 3 biological replicates. Two-tailed unpaired *t*-test. (G) Flow cytometric analysis of mitochondrial ROS in primary hMSCs isolated from a 79-year-old individual transduced with lentiviruses expressing Luc or 4E-BP1. Dashed lines indicate MFI of Luc or 4E-BP1 expressing hMSCs. Statistical data are presented as means ± SEM. *n* = 3 biological replicates. Two-tailed unpaired *t*-test. (H) SA-β-gal staining of primary hMSCs isolated from a 79-year-old individual transduced with lentiviruses expressing Luc or 4E-BP1. Scale bars, 100 μm. Statistical data are presented as means ± SEM. *n* = 3 biological replicates. Two-tailed unpaired *t*-test. (I) Clonal expansion assay in primary hMSCs isolated from a 79-year-old individual transduced with lentiviruses expressing Luc or 4E-BP1. Statistical data are presented as means ± SEM. *n* = 4 biological replicates. Two-tailed unpaired *t*-test.

To further evaluate the potential geroprotective role of 4E-BP1 via regulating mitochondrial homeostasis in hMSCs, we re-introduced 4E-BP1 into *EIF4EBP1*^−/−^ hMSCs using a lentiviral vector expressing 4E-BP1 (Fig. 6A). The re-expression of 4E-BP1 not only increased the protein level of UQCRC2 (Fig. 6B) but also partially rescued the mitochondrial function in *EIF4EBP1*^−/−^ hMSCs, as indicated by decreased mitochondrial ROS levels (Fig. 6C). Moreover, the re-expression of 4E-BP1 alleviated hMSC senescence, as revealed by the declined number of SA-β-gal-positive cells and augmented colony formation ability (Fig. 6D and 6E).

Given that 4E-BP1 was downregulated in primary hMSCs isolated from aged individuals (Fig. 1B), we examined whether 4E-BP1 overexpression could rejuvenate physiologically aged human stem cells (Fig. 6A). Indeed, 4E-BP1 overexpression increased protein levels of UQCRC2 in physiologically aged hMSCs (Fig. 6F). In addition, 4E-BP1 overexpression decreased mitochondrial ROS levels and attenuated senescence phenotypes, as manifested by the reduced number of SA-β-gal-positive cells and enhanced colony formation ability in aged primary hMSCs (Fig. 6G–I). Taken together, these findings indicate that 4E-BP1 exerts a geroprotective role in hMSCs at least in part by enhancing the expression levels of mitochondrial OXPHOS complex III core component, UQCRC2, and restoring mitochondrial fitness (Fig. S2K).

## Discussion

In this study, we discovered a geroprotective role of 4E-BP1 in hMSCs via promoting mitochondrial fitness. First, we observed that the expression of 4E-BP1 was downregulated in replicatively and physiologically senescent hMSCs and that the depletion of 4E-BP1 accelerated hMSC senescence. In addition, we found that 4E-BP1 was partially localized inside the mitochondria, indispensable for maintaining mitochondrial homeostasis via stabilizing the mitochondrial OXPHOS complex III component UQCRC2. Importantly, overexpression of 4E-BP1 increased the protein level of UQCRC2, leading to improved mitochondrial fitness and alleviated hMSC senescence. Accordingly, to our knowledge, our findings demonstrate a novel role of 4E-BP1 in the alleviation of human stem cell senescence at least in part through modulating mitochondrial homeostasis.

Although previous results have indicated that 4E-BP1 is associated with aging, most of those studies were carried out in model organisms, including *Drosophila* and rodents (Carvalho *et al.*, 2017; Kang *et al.*, 2017; Le Bacquer *et al.*, 2019). For example, 4E-BP was reported to be necessary for maximal lifespan extension upon DR in *Drosophila* (Zid *et al.*, 2009). In addition, overexpression of constitutively active 4E-BP in the muscle of *Drosophila* extends their median and maximum life spans (Demontis and Perrimon, 2010). Likewise, transgenic expression of 4E-BP1 in male mice alleviates metabolic disorder and aging-induced obesity (Tsai *et al.*, 2016a) while the deficiency of 4E-BP1 and 4E-BP2 accelerates mouse embryonic fibroblast senescence (Petroulakis *et al.*, 2009). Here, we have generated 4E-BP1-deficient human stem cells via CRISPR/Cas9-based strategy and revealed a geroprotective function of 4E-BP1 in hMSCs. Our findings together with previous reports suggest a crucial role of 4E-BP1 in counteracting senescence/aging that is evolutionarily conserved from model organisms (e.g., *Drosophila* and rodent) to human stem cells, thus providing promising potentials for clinical translation, especially in the interventions of aging and age-related diseases (Petroulakis *et al.*, 2009; Demontis and Perrimon, 2010; Tsai *et al.*, 2016b; Wang *et al.*, 2022).

Mitochondrial dysfunction is regarded as a marker and an inducer of stem cell aging (Oh *et al.*, 2014; Payne and Chinnery, 2015). Mitochondria provide necessary energy and substrates for cell metabolism and growth through the respiration process (Xu *et al.*, 2013; Zong *et al.*, 2016; Vakifahmetoglu-Norberg *et al.*, 2017; Spinelli and Haigis, 2018). However, abnormal mitochondria lead to reduced energy supply and increased ROS release, thus contributing to the disrupted mitochondrial homeostasis and induction of cellular senescence (Balaban *et al.*, 2005; Oh *et al.*, 2014; Ziegler *et al.*, 2015). A previous study has revealed that 4E-BP1 homolog in *Drosophila* positively mediates mitochondrial activity by regulating the translation of nuclear-encoded mitochondrial genes (Zid *et al.*, 2009), implying an indirect manner by which 4E-BP1 modulates mitochondrial activity. In this study, we identified a critical role of 4E-BP1 in the maintenance of mitochondrial homeostasis, as knockout of 4E-BP1 caused mitochondrial abnormalities, energy metabolism disorders, and ROS accumulation in hMSCs. Different from the reported function of 4E-BP1 in modulating mitochondrial activity via translational regulation, we found that 4E-BP1 interacted with and stabilized the OXPHOS complex III component UQCRC2, thus rescuing mitochondrial fitness and counteracting hMSC senescence. Therefore, our study provides a more direct model for 4E-BP1 in the regulation of mitochondrial energy metabolism, which helps reveal the potential crosstalk between mTOR pathway-related nutrient awareness and mitochondria-mediated energy metabolism, in the process of human stem cell aging. Although previous studies hinted a clue that UQCRC2 may be degraded through mitophagy or ubiquitin–proteasome pathway (Shukla *et al.*, 2013; Moskal *et al.*, 2020), the role of 4E-BP1 in this process remains to be further investigated.

As a core subunit of mitochondrial respiratory complex III, UQCRC2 is indispensable for the assembly of the complex III. Mutations in UQCRC2 severely decrease the complex III level (Miyake *et al.*, 2013; Tucker *et al.*, 2013; Gaignard *et al.*, 2017), which in turn contributes to impaired cellular homeostasis such as the inhibited proliferation of endothelial cells (Andrade and Potente, 2019). Consistently, here we found that UQCRC2 knockdown also led to compromised proliferation accompanied by accelerated senescence in hMSCs, mimicking phenotypes observed in 4E-BP1-deficient cells. Moreover, we detected reduced levels of other components (e.g. UQCRFS1) in complex III upon UQCRC2 depletion. Supporting our findings, previous studies have reported that mitochondrial respiratory chain enzymes associate interdependently in a super complex, and defects in a single component usually cause impairments in the whole complex (Protasoni *et al.*, 2020).

Notably, different from what we observed in hMSCs, 4E-BP1 depletion did not adversely affect the genomic stability, self-renewal, and pluripotency of hESCs, suggesting a more refined homeostasis regulation system with cell-type specificity in hESCs. Whether hESCs may exploit other different mitochondrial fitness-maintaining mechanisms remains to be investigated. These findings further raise questions about how machineries of translation and energy metabolism coordinate to finely regulate the homeostasis in distinct types of human stem cells.

Taken together, we have discovered a novel function of 4E-BP1 as a stabilizer of mitochondrial homeostasis and a geroprotector for antagonizing hMSC senescence. Importantly, we report a previously uncharacterized mechanism by which 4E-BP1 maintains mitochondrial homeostasis and functions via stabilizing a core component of mitochondrial OXPHOS complex III. These findings offer new insights into the role of 4E-BP1 in regulating human stem cell aging and provide potential targets against aging and aging-associated diseases via the modulation of mitochondrial fitness.

## Materials and methods

### Animal experiments

Animal experiments were performed under the approval of the Chinese Academy of Sciences Institutional Animal Care and Use Committee. *In vivo* hMSC implantation assay was conducted as previously reported (Bi *et al.*, 2020). Briefly, 1 × 10^6^*EIF4EBP1*^+/+^ or *EIF4EBP1*^−/−^ hMSCs expressing luciferase (Luc) were implanted into the tibialis anterior (TA) muscle of male nude mice, and Luc signal was detected with an *in vivo* imaging system (IVIS; Xenogen, Caliper) at day 1, day 3 and day 5 after implantation.

### Cell culture

*EIF4EBP1* ^+/+^ (H9 hESCs, WiCell Research Institute) and *EIF4EBP1*^−/−^ hESCs were maintained on feeder layers of mitomycin C-inactivated mouse embryonic fibroblasts (MEFs) in hESC culture medium CDF12 [DMEM/F12 (Thermo Fisher Scientific), 20% Knockout Serum Replacement (Thermo Fisher Scientific), 0.1 mmol/L non-essential amino acids (NEAA, Thermo Fisher Scientific), 2 mmol/L GlutaMAX (Thermo Fisher Scientific), 1% penicillin/streptomycin (Thermo Fisher Scientific), 55 μmol/L β-mercaptoethanol (Thermo Fisher Scientific), and 10 ng/mL bFGF (Joint Protein Central)], or on Matrigel in mTeSR medium. Primary hMSCs and hESC-derived hMSCs were cultured in hMSC culture medium [αMEM with GlutaMAX (Thermo Fisher Scientific), 10% fetal bovine serum (FBS, Gemcell), 1% penicillin/streptomycin and 1 ng/mL bFGF]. No mycoplasma contamination was observed during cell culture.

### Generation of *EIF4EBP1*^−/−^ hESCs

CRISPR/Cas9-mediated gene editing was performed as previously described (Chu *et al.*, 2022). In brief, sgRNA targeting exon 1 of *EIF4EBP1* was designed using an online system as previously reported (Song *et al.*, 2022), and then cloned into pCAG-mCherry-sgRNA vector (Addgene, #87110). *EIF4EBP1*^+/+^ hESCs cultured on Matrigel-coated plates (Corning) were treated with ROCK inhibitor (Y-27632, TOCRIS) for 10 h before electroporation. Then, pCAG-mCherry-*EIF4EBP1*-sgRNA vector and pCAG-1BPNLS-Cas9-1BPNLS-2AGFP vector (Addgene, #87109) were simultaneously electroporated into *EIF4EBP1*^+/+^ hESCs using a 4D-Nucleofector (Lonza). After electroporation, hESCs were seeded on Matrigel-coated plates and cultured in mTeSR medium for 48 h, with ROCK inhibitor addition on the first day. Subsequently, GFP and mCherry double-positive cells were isolated with a fluorescence-activated cell sorting (FACS) System (BD FACS Aria) and seeded on MEF feeders with hESC culture medium. Emerging clones were manually picked out for genomic DNA extraction and subsequent PCR and sequencing. sgRNA sequence used for gene editing and primers used for PCR and sequencing are listed in Table S3.

### Generation of *EIF4EBP1*^+/+^ and *EIF4EBP1*^−/−^ hMSCs

*EIF4EBP1* ^+/+^ and *EIF4EBP1*^−/−^ hMSCs were differentiated from hESCs as previously described (Fu *et al.*, 2019). Briefly, *EIF4EBP1*^+/+^ and *EIF4EBP1*^−/−^ hESCs were dissociated into embryoid bodies and then seeded on Matrigel-coated plates in hMSC differentiation medium [αMEM with GlutaMAX, 10% FBS, 1% penicillin/streptomycin, 10 ng/mL bFGF, and 5 ng/mL TGFβ (HumanZyme)]. After around 10 days, the confluent fibroblast-like cells were transferred into hMSC culture medium for further culture. Cells were harvested at 90% confluence, and hMSCs were sorted by a FACS System (BD FACS Influx) via selecting CD73, CD90, and CD105 triple-positive cells, which were further characterized by hMSC-positive markers, such as CD166, CD29, CD44, CD13, and HLA, as well as hMSC-irrelevant markers, such as CD34, CD43, CD45, CD14, CD19, CD164, and PDPN. Finally, the differentiation capacity of hESC-derived hMSCs toward osteoblasts or adipocytes was evaluated by Von Kossa staining and Oil Red O staining, respectively. The primary antibodies used for FACS were as follows: anti-CD73 (BD 550257, 1:200), anti-CD90 (BD 555595, 1:200), anti-CD105 (eBioscience 17-1057-41, 1:200), anti-CD34 (BD 555822, 1:200), anti-CD43 (BD 560198, 1:200), anti-CD45 (BD 555482, 1:200), anti-CD166 (Biolegend 343903, 1:200), anti-CD29 (Biolegend 303007, 1:200), anti-CD44 (BD 555478, 1:200), anti-CD13 (Biolegend 301705, 1:200), anti-HLA-ABC (BD 560168, 1:100), anti-CD14 (BD 555398, 1:200), anti-CD19 (BD 555415, 1:200), anti-CD164 (Biolegend 324805, 1:200), and anti-PDPN (eBioscience 17-9381-42, 1:200).

### Isolation and culture of primary hMSCs

Primary hMSCs were isolated from different individuals under the approval of the ethics committee as previously reported (Liang *et al.*, 2021). In brief, the gingiva tissues were processed for digestion in TrypLE™ Express Enzyme (Thermo Fisher Scientific) plus Dispase IV (Thermo Fisher Scientific) at 37°C for 30 min, filtered with a 70-μm cell strainer (Falcon), and then centrifuged at 1,000 rpm for 5 min. Subsequently, the pellets were resuspended in hMSC culture medium and seeded on a gelatin-coated 6-well plate (Corning) for further culture.

### Plasmid construction

To generate FLAG-tagged 4E-BP1 and 3×FLAG-tagged 4E-BP1 expression plasmids, *EIF4EBP1* cDNA was generated from *EIF4EBP1*^+/+^ hMSCs via reverse-transcription (RT) PCR amplification and then cloned into the pLE4 empty vector (a kind gift from Dr. Tomoaki Hishida) using a NovoRec^®^ Plus One-Step PCR Cloning Kit (Novoprotein) according to the manufacturer’s instructions. For lenti-CRISPR/Cas9 mediated UQCRC2 knockout assay, sgRNA targeting *UQCRC2* (sg-*UQCRC2*) was cloned into the lentiCRISPRv2 vector (Addgene, #52961) as previously described (Zhang *et al.*, 2022). shRNA targeting *UQCRB* (sh-*UQCRB*) was cloned into the pLVTHM vector (Addgene, #12247). Primers used for plasmid construction are listed in Table S3.

### Lentivirus packaging

Lentivirus packaging was conducted as previously described with some modifications (Hu *et al.*, 2020). Briefly, 4E-BP1 expression, sg-*UQCRC2* or sh-*UQCRB* plasmids were transfected into HEK293T cells together with lentiviral packing vectors including psPAX2 (Addgene, #12260) and pMD2.G (Addgene, #12259). Then, the cell culture medium was collected at 32 h and 56 h post-transfection by ultracentrifugation at 19,400 rpm at 4°C for 2 h, and the pellets (lentiviral particles) were gently resuspended in Opti-MEM (Thermo Fisher Scientific) medium for lentiviral transduction of hMSCs.

### Western blot

In brief, cell pellets were resuspended in 1× SDS lysis buffer (62.5 mmol/L Tris-HCl (pH 6.8), 2% (wt/vol) SDS), boiled at 105°C for 10 min, and then processed for protein concentration measurement with a BCA Protein Assay Kit (Ding Guo Chang Sheng). Next, 20 μg protein was prepared for SDS-PAGE separation and electrotransferred to PVDF membranes (Millipore). After blocking with 5% nonfat milk (BBI Life Sciences) for 1 h at room temperature (RT), the membranes were incubated with primary antibodies overnight at 4°C, and then incubated with corresponding secondary antibodies conjugated with horseradish peroxidase (HRP) for 1 h at RT, and then, washed in TBST (20 mmol/L Tris-HCl (pH 7.6), 137 mmol/L NaCl, 0.2% Tween-20) for three times. Images were captured with a ChemiDoc XRS+ System (Bio-Rad) and band intensity was determined using ImageJ. The primary antibodies used for western blot analysis were as follows: anti-4E-BP1 (CST 9644, 1:3,000), anti-β-actin (Santa sc-69879, 1:3,000), anti-OXPHOS (Abcam ab110411, 1:1,000), anti-UQCRC2 (Santa sc-390378, 1:500), anti-UQCRB (Abcam ab190360, 1:1,000), anti-UQCRFS1 (Santa sc-271609, 1:200), anti-p-mTOR (CST 2971, 1:1,000), and anti-FLAG (Sigma F1804, 1:1,000).

### Immunofluorescence staining

For immunofluorescence analysis, cells seeded on coverslips (Thermo Fisher Scientific) were fixed with 4% paraformaldehyde (PFA) for 20 min, permeabilized with 0.4% Triton X-100 in PBS for 20 min, and then blocked with 10% donkey serum (Jackson ImmunoResearch) for 1 h at RT. After blocking, cells were incubated with primary antibodies at 4°C overnight, rinsed by PBS, and then incubated with corresponding secondary antibodies at RT for 1 h, protected from light. Hoechst 33342 (Thermo Fisher Scientific) was used for nuclear DNA staining. Images were obtained with a Leica SP5 Confocal System and Zeiss Confocal System LSM900. The primary antibodies used for immunofluorescence analysis were as follows: anti-Ki67 (ZSGB-BIO ZM0166, 1:1,000), anti-γH2AX (Millipore 05-636, 1:500), anti-53BP1 (Bethyl Laboratories A300-273A, 1:500), anti-COXIV (CST 4850, 1:100), anti-OCT3/4 (Santa sc-5279, 1:100), anti-SOX2 (Santa sc-17320, 1:100), and anti-NANOG (Abcam ab21624, 1:100).

### Senescence-associated β-galactosidase (SA-β-gal) staining

SA-β-gal staining was performed as previously described (Wu *et al.*, 2018). In brief, cells were fixed in fixation buffer (2% formaldehyde and 0.2% glutaraldehyde) for 5 min at RT and stained with fresh SA-β-gal staining solution [150 mmol/L NaCl, 2 mmol/L MgCl_2_, 40 mmol/L citric acid/Na phosphate buffer, 5 mmol/L K_3_(Fe[CN]_6_), 5 mmol/L K_4_(Fe[CN]_6_), 1 mg/mL X-gal (AMRESCO, 0428-25G)] at 37°C overnight in the dark. Images were captured using a digital camera combined with an optical microscope (Olympus) and the percentage of SA-β-gal-positive cells was determined with ImageJ.

### Clonal expansion assay

Clonal expansion assay was conducted as previously described (Geng *et al.*, 2019). In brief, 2,000 cells were seeded in each well of a 12-well plate (Corning) and cultured for around 10 days. Then, cells were washed with PBS, fixed with 4% PFA for 20 min, and stained with 10% crystal violet (Biohao Biotech) for 1 h at RT. Pictures were captured using a scanner (EPSON, V370). The relative cell density was determined using ImageJ.

### DNA and RNA detection and quantification

For DNA analysis, genomic DNA was extracted using a Blood/Cell/Tissue Genomic DNA Extraction Kit (TIANGEN) and PCR amplification was performed using PrimeSTAR DNA Polymerase Kit (TaKaRa). Primers used for PCR amplification are listed in Table S3.

For RNA analysis, total RNA was extracted using TRIzol Reagent (Thermo Fisher Scientific) and processed for reverse transcription using a GoScript^TM^ Reverse Transcription System (Promega). Then, quantitative PCR (qPCR) was conducted using THUNDERBIRD^TM^ SYBR^®^ qPCR Mix (TOYOBO) with a CFX384 Real-Time PCR Detection System (Bio-Rad). Primers used for qPCR analysis are listed in Table S3.

### Transmission electron microscopy (TEM)

Transmission electron microscopy was performed as previously reported (Diao *et al.*, 2021). Briefly, *EIF4EBP1*^+/+^ and *EIF4EBP1*^−/−^ hMSCs were collected using TrypLE™ Express Enzyme, pelleted by centrifugation at 500 ×*g* for 5 min, fixed with 2.5% (vol/vol) glutaraldehyde at 4°C overnight, and post-fixed with 1% (wt/vol) osmium tetraoxide at 4°C for 2 h. Subsequently, dehydration was completed through a graded series of ethanol, followed by infiltration in a mixture of acetone and SPI-PON812 resin. Ultrathin sections were obtained using a Leica EM UC6 Ultramicrotome, and then stained with uranyl acetate and lead citrate. A Spirit Transmission Electron Microscope (FEI Company) at 100 kV was used for imaging. Then, mitochondrial numbers were counted in an intact cell, and a total of 30 cells were calculated in each sample. Subsequently, abnormal mitochondrial numbers were counted in each sample to determine the relative percentage of abnormal mitochondria. Definition of abnormal mitochondria follows previous reports (Feher *et al.*, 2006; Daum *et al.*, 2013; Szeto and Liu, 2018). In brief, the mitochondria with obvious disruption of mitochondrial cristae structure and inner membrane vesiculates were defined as abnormal mitochondria, and the relative percentage of abnormal mitochondria from 40 images for each sample was quantified. The numbers of abnormal and total mitochondria were determined using ImageJ-based Fiji software.

### Measurement of total ROS and mitochondrial ROS levels

hMSCs were digested by TrypLE™ Express Enzyme into single cells, and then stained by CM-H_2_DCFDA (Invitrogen C6827, 1:2,000) or MitoSOX™ Red (Invitrogen M36008, 1:1,000) and incubated at 37°C in a CO_2_ incubator for 15 min. The cells were then washed with PBS and detected by FACS. Data were analyzed using Flow Jo software. The median was used for the evaluation of mean fluorescence intensity (MFI) in *EIF4EBP1*^+/+^ and *EIF4EBP1*^−/−^ hMSCs.

### Measurement of cellular H_2_O_2_ level

The H_2_O_2_ level in hMSCs was measured using Amplex Red Hydrogen Peroxide/Peroxidase Assay Kit (Invitrogen A22188) according to the manufacturer’s instructions. In brief, hMSCs were lysed on ice using lysis buffer, then the supernatants were obtained after centrifugation. The resultant supernatants were spotted on the bottom of an opaque clean 96-well plate and incubated with a working solution at RT for 30 min in the dark. After the reaction, the samples were detected by a microplate reader (BioTek spectrophotometer) immediately. The detection parameters were set as follows: excitation, 530 nm; emission, 590 nm. Each sample was measured in triplicate. Samples were estimated by BCA protein quantification to correct for differences in loading.

### Seahorse assay

Mitochondrial respiration function of cells was evaluated by measuring the oxygen consumption rate (OCR) before and after the addition of respiration regulators. On the day prior to the experiment, hMSCs were seeded into Seahorse XF cell culture microplates at a density of 2 × 10^4^ per well. Then the Seahorse XF Analyzer was powered on to warm up. Finally, the Seahorse XF sensor cartridge was incubated by calibration solution overnight at 37°C in a CO_2_-free incubator.

Before the start of the experiment, Seahorse assay medium (Seahorse XF basal medium containing 10 mmol/L glucose, 1 mmol/L sodium pyruvate, and 2 mmol/L glutamine) was prepared and warmed in a 37°C water bath. After culture for 36 h, Seahorse XF cell culture microplate was washed twice using Seahorse assay medium, and then incubated at 37°C in a CO_2_-free incubator for 1 h with 180 μL Seahorse assay medium per well. Respiration regulators oligomycin (1 μmol/L), FCCP (1 μmol/L), and rotenone/antimycin A (0.5 μmol/L) were prepared and added into the pre-calibrated Seahorse XF sensor cartridge. The experiment was run and analyzed using the software “Wave”. Protein abundance was usually measured for subsequent data normalization (Silva *et al.*, 2013; Yao *et al.*, 2019; McCrimmon *et al.*, 2020; Burgstaller *et al.*, 2021). Similar to the normalization method, we seeded same number of cells for each sample in this experiment and detected comparable protein amounts between *EIF4EBP1*^+/+^ and *EIF4EBP1*^−/−^ hMSCs (at both EP and LP) after 36 h culture and presented the original OCR values of each sample in this study.

### CNV analysis

The genomic DNA of hESCs was extracted from 1 × 10^6^ cells with a Blood/Cell/Tissue Genomic DNA Extraction Kit (TIANGEN). Subsequently, DNA quality control, library preparation, and sequencing on Illumina HiSeq X Ten platforms were performed by Novogene Bioinformatics Technology Co. Ltd. Data analysis was performed using published R packages as previously described (Zhao *et al.*, 2022). In brief, we trimmed raw reads with TrimGalore. Then, the clean reads were mapped to the human hg19 genome. The mapped reads were further filtered using samtools and Picard software. The remaining reads were counted for each 500-kb window using read counter function in HMMcopy_utils. The R/Bioconductor package HMMcopy (v1.26.0) was used to correct copy number, GC content, and mappability.

### RNA-seq data analysis

RNA-seq data processing was performed as previously described (Li *et al.*, 2022). In brief, low-quality reads and adaptors were first trimmed using TrimGalore (v0.4.4_dev). The remaining clean reads were mapped to the UCSC human hg19 genome using HISAT2 software (v2.2.1). Reads on each annotated gene were counted using featureCounts (v2.0.1). Differentially expressed genes (DEGs) were calculated using DESeq2, if their adjusted *P*-value < 0.05 and absolute average log_2_ ratio > 0.58. GO term enrichment analysis was performed using Metascape (Zhou et al., 2019). The DEGs are listed in Table S1.

### Co-immunoprecipitation (Co-IP) analysis

The Co-IP assay was performed as previously reported (Diao *et al.*, 2021). HEK293T cells were transfected with plasmids expressing FLAG-Luc or 3×FLAG-4E-BP1. To be noted, the 3×FLAG-4E-BP1 was used in this assay due to the weak enrichment of FLAG-4E-BP1 by anti-FLAG Affinity Gel (Sigma A2220). At 48 h after transfection, cells were lysed with CHAPS buffer [40 mmol/L HEPES, 0.3% CHAPS (Sigma V900480), 120 mmol/L NaCl, 1 mmol/L EDTA (pH 7.5), and 1× complete protease inhibitor cocktail (Roche), 1× PMSF], and spun on a rotator at 4°C for 2 h. Lysates were centrifuged at 14,800 rpm and at 4°C for 30 min. The supernatants were collected carefully (to avoid taking the top layer of the oil phase). The supernatants were measured by BCA protein quantification, then adjusted to the same concentration and volume according to the quantitative results. After adjustment, a part of the supernatants was obtained as input. Anti-FLAG Affinity Gel was then added to the remaining supernatants, followed by incubation on a rotator at 4°C overnight. Immunocomplexes were obtained after centrifuging at 3,000 rpm at 4°C for 1 min and the pellets were washed with CHAPS buffer five times for 5 min each time. FLAG peptides were added to the pellets for competitive elution. The samples were spun on a rotator at 4°C for 2 h. The supernatants were obtained after elution, mixed with 1× SDS protein loading buffer, and then boiled at 105°C for 10 min. Subsequently, the obtained samples were further used for western blot analysis.

### Protein stability analysis

hMSCs were treated with cycloheximide (CHX, Sigma) at a final concentration of 20 μg/mL for 0, 3, 6, 9, and 12 h, and then lysed with 1× SDS for subsequent western blot analysis. Due to the different expression levels of the target proteins UQCRC2, UQCRB, and UQCRFS1 in *EIF4EBP1*^+/+^ and *EIF4EBP1*^−/−^ hMSCs, images with relatively shorter exposure time were collected for quantification of *EIF4EBP1*^+/+^ hMSCs, while images with relatively longer exposure time were collected for quantification of *EIF4EBP1*^−/−^ hMSCs.

### Polysome profiling analysis

Polysome profiling assay was performed as previously reported (Gandin *et al.*, 2014). hMSCs were treated with cycloheximide (CHX, Sigma) (at a final concentration of 100 μg/mL) for 5 min, washed with PBS containing 100 μg/mL CHX, and disassociated into single cells by TrypLE™ Express Enzyme. The collected hMSCs were washed with PBS and then lysed with 425 μL hypotonic buffer [5 mmol/L Tris-HCl (pH 7.5), 2.5 mmol/L MgCl_2_, 1.5 mmol/L KCl, and 1× protease inhibitor cocktail (EDTA-free)]. Then, the lysates were supplemented with 5 μL of 10 mg/mL CHX, 1 μL of 1 mol/L DTT, 100 units of RNase inhibitor, and vortexed for 5 s, followed by the addition of 25 μL 10% Triton X-100 and 25 μL 10% sodium deoxycholate, and then vortexed for another 5 s. Subsequently, the lysates were centrifuged at 16,000 × *g* at 4°C for 7 min, and the supernatants were collected. After adjusting the absorbance of cell lysates to OD 260 nm, 10% of the lysates as input were diluted with RNase-free water to 750 μL, and added with the same volume of TRIzol, then stocked in liquid nitrogen. The remaining lysates were loaded into 10%–50% gradient sucrose tubes, and then centrifuged at 36,000 rpm at 4°C for 2 h. Gradients were then isolated by a Density Gradient Fractionation System. The absorbance of all samples at 254 nm was constantly monitored by the ISCO monitor. Subsequently, samples distributed in different gradients were constantly collected by a collector. The collected fractions were added with an equal volume of TRIzol, soaked in liquid nitrogen immediately, and then stored at −80°C. In order to further analyze the translation state, the RNAs were extracted, and the cDNAs were obtained by reverse transcription. The abundance of the mRNAs of target genes in every gradient fraction was detected by qRT-PCR. The results were analyzed referring to a previous report (Panda *et al.*, 2017). Finally, the distribution of target mRNAs from different sucrose density gradients was analyzed to reflect the translation state.

### Quantitative proteomic analysis

hMSCs were digested into single cells by TrypLE™ Express Enzyme and lysed by lysis buffer (8 mol/L urea/0.1 mol/L Tris-HCl, pH 8.0) containing 1× protease inhibitor cocktail (Roche). Then the total proteins were extracted after centrifugation and digested into peptides. Subsequently, the peptides from each group were labeled with TMT 6plex^®^ (Thermo Fisher Scientific) according to the instructions. After labeling, the samples were fractionated by HPLC System. Then, the LC-MS/MS analysis was performed with a Q Exactive mass spectrometer (Thermo Fisher Scientific) and data was processed using Proteome Discovery (v2.2.0.388).

Differentially expressed proteins (DEPs) between *EIF4EBP1*^−/−^ versus *EIF4EBP1*^+/+^ hMSCs were filtered after statistical analysis using unpaired Student’s *t*-test, with the cutoff for *P*-value < 0.05 and absolute average log_2_ ratio > 0.2. GO term enrichment analysis was performed using Metascape. The network of enriched terms was generated with Metascape and visualized with Cytoscape (v3.8.2). Gene set enrichment analysis was performed using Gene Set Enrichment Analysis (GSEA) software (v4.0.3). The DEPs are listed in Table S2.

### Statistical analysis

Statistical data are presented as means ± SEM or means ± SD. GraphPad Prism 8 software is used to conduct a two-tailed unpaired *t*-test. *P* < 0.05 was considered statistically significant. *P*-values are presented in indicated figures.

## Supplementary data

The online version contains supplementary material available at https://doi.org/10.1093/procel/pwac037.

pwac037_suppl_Supplementary_Material

pwac037_suppl_Supplementary_Table_S1

pwac037_suppl_Supplementary_Table_S2

pwac037_suppl_Supplementary_Table_S3

## Data Availability

The high-throughput sequencing data including RNA-seq and whole-genome sequencing (WGS) generated in this study have been deposited in the Genome Sequence Archive (GSA) in the National Genomics Data Center, Beijing Institute of Genomics (China National Center for Bioinformation) of the Chinese Academy of Sciences under the accession number HRA002275. The proteomic data have been deposited in the ProteomeXchange Consortium via the PRIDE partner repository with the dataset identifier PXD033301. Other data or materials generated in this study are available from the corresponding authors upon reasonable request. All the custom codes are available from the corresponding authors upon reasonable request.

## Abbreviations

4E-BP1: eukaryotic translation initiation factor 4E-binding protein 1
4E-BP2: eukaryotic translation initiation factor 4E-binding protein 2
CHX: cycloheximide
CNV: copy number variation
Co-IP: co-immunoprecipitation
DEGs: differentially expressed genes
DEPs: differentially expressed proteins
DR: dietary restriction
eIF4E: eukaryotic translation initiation factor 4E
EP: early passage
FACS: fluorescence-activated cell sorting
FBS: fetal bovine serum
GO: Gene Ontology
GSEA: Gene Set Enrichment Analysis
hESCs: human embryonic stem cells
hMSCs: human mesenchymal stem cells
HRP: horseradish peroxidase
IVIS: *in vivo* imaging system
LP: late passage
Luc: luciferase
MEFs: mouse embryonic fibroblasts
MFI: mean fluorescence intensity
mTORC1: mechanistic target of rapamycin complex 1
NEAA: non-essential amino acids
NHEJ: nonhomologous end-joining
OCR: oxygen consumption rates
OXPHOS: oxidative phosphorylation
qRT-PCR: quantitative reverse-transcription PCR
RNA-seq: RNA-sequencing
ROS: reactive oxygen species
SASP: senescence-associated secretory phenotype
SA-β-gal: senescence-associated β-galactosidase
TA: tibialis anterior
WGS: whole-genome sequencing
