# Supplementary material for: 4E-BP1 counteracts human mesenchymal stem cell senescence via maintaining mitochondrial homeostasis
Source: Protein Cell. 2022 Aug 23;14(3):202–16. doi: 10.1093/procel/pwac037 (PMC10098039; doi:10.1093/procel/pwac037)

Supplemental Materials

Supplemental Figure Legends

Figure S1. Generation of *EIF4EBP1*$^{-/-}$ hESCs by CRISPR/Cas9-mediated gene editing. (A) Western blot analysis of 4E-BP1 in *EIF4EBP1*$^{+/+}$ and *EIF4EBP1*$^{-/-}$ hESCs. β-actin was used as loading control. (B) Karyotype analysis of *EIF4EBP1*$^{-/-}$ hESCs. (C) The copy number variation (CNV) analysis in *EIF4EBP1*$^{+/+}$ and *EIF4EBP1*$^{-/-}$ hESCs. (D) Detection of the top 6 predicted off-target sites in *EIF4EBP1*$^{-/-}$ hESCs. (E) Immunofluorescence staining of pluripotency markers, including OCT4, SOX2 and NANOG in *EIF4EBP1*$^{+/+}$ and *EIF4EBP1*$^{-/-}$ hESCs. Scale bars, 10 μm. (F) Semi-quantitative PCR (semi-qPCR) analysis of the expression levels of pluripotency markers, including *SOX2*, *OCT4* and *NANOG* in *EIF4EBP1*$^{+/+}$ and *EIF4EBP1*$^{-/-}$ hESCs. *18S rRNA* was used as a loading control. (G) Cell cycle analysis of *EIF4EBP1*$^{+/+}$ and *EIF4EBP1*$^{-/-}$ hESCs. Statistical data are presented as means ± SEM. n = 3 biological replicates. Two-tailed unpaired t-test.

Figure S2. Generation of *EIF4EBP1*$^{+/+}$ and *EIF4EBP1*$^{-/-}$ hMSCs. (A) Flow cytometric analysis of classic markers for hMSCs, including CD73, CD90 and CD105, as well as irrelevant markers for hMSCs, including CD34, CD43 and CD45 in *EIF4EBP1*$^{+/+}$ and *EIF4EBP1*$^{-/-}$ hMSCs. (B) Ratio analysis of cells positive for surface antigen markers for hMSCs, including CD166, CD29, CD44, CD13 and HLA, or irrelevant markers for hMSCs, including CD14, CD19, CD164, and PDPN, in *EIF4EBP1*$^{+/+}$ and *EIF4EBP1*$^{-/-}$ hMSCs. Statistical data are presented as means ± SD. n = 3 technical replicates. (C-D) Characterization of the differentiation potentials of *EIF4EBP1*$^{+/+}$ and *EIF4EBP1*$^{-/-}$ hMSCs (P4) into osteoblasts (C) and adipocytes (D). Von Kossa staining was used to evaluate osteogenesis. Scale bars, 100 μm. The relative Von Kossa-positive area is quantified as fold changes of areas in *EIF4EBP1*$^{-/-}$ cells compared with that in *EIF4EBP1*$^{+/+}$ cells and presented as means ± SEM. n = 3 biological replicates. Two-tailed unpaired t-test. Oil Red O staining was used to evaluate adipogenesis. Scale bars, 100 μm. The relative absorbance of Oil Red O is quantified as fold changes of absorbance value in *EIF4EBP1*$^{-/-}$ cells compared with that in *EIF4EBP1*$^{+/+}$ cells and presented as means ± SEM. n = 3 biological replicates. Two-tailed unpaired t-test. (E) SA-β-gal staining of *EIF4EBP1*$^{+/+}$ and *EIF4EBP1*$^{-/-}$ hMSCs (P4). Scale bars, 100 μm. Statistical data are presented as means ± SEM. n = 3 biological replicates. Two-tailed unpaired t-test. (F) Clonal expansion assay in *EIF4EBP1*$^{+/+}$ and *EIF4EBP1*$^{-/-}$ hMSCs (P4). Statistical data are presented as means ± SEM. n = 3 biological replicates. Two-tailed unpaired t-test. (G) Immunofluorescence staining of Ki67 in *EIF4EBP1*$^{+/+}$ and *EIF4EBP1*$^{-/-}$ hMSCs (P4). Arrows indicate Ki67-positive cells. Scale bars, 10 μm. Statistical data are presented as means ± SEM. n = 3 biological replicates. Two-tailed unpaired t-test. (H) Cell cycle analysis of *EIF4EBP1*$^{+/+}$ and *EIF4EBP1*$^{-/-}$ hMSCs (P4). Statistical data are presented as means ± SEM. n = 3 biological replicates. Two-tailed unpaired t-test. (I) Heatmap showing the Euclidian distance between replicates among the indicated groups of RNA-seq data of *EIF4EBP1*$^{+/+}$ and *EIF4EBP1*$^{-/-}$ hMSCs at EP (P4) and LP (P8). (J) Principal component

analysis showing high reproducibility of quantitative proteomic data between replicates in *EIF4EBP1*$^{+/+}$ and *EIF4EBP1*$^{-/-}$ hMSCs (P4). (K) A model describing the role of 4E-BP1 as a stabilizer of UQCRC2 in *EIF4EBP1*$^{+/+}$ hMSCs. In *EIF4EBP1*$^{+/+}$ hMSCs, 4E-BP1 potentially interacts with and stabilizes UQCRC2, thus promoting the stability of mitochondrial respiration complex III, maintaining mitochondrial homeostasis, and counteracting cellular senescence. 4E-BP1 deficiency in hMSCs results in decreased expression of UQCRC2, destabilization of mitochondrial complex III, disrupted mitochondrial structure and function, excessive accumulation of ROS, and accelerated cellular senescence. Schematic diagram was created with BioRender.com.

Supplemental Table Legends

Table S1. Differentially expressed genes (DEGs) between *EIF4EBP1*$^{-/-}$ and *EIF4EBP1*$^{+/+}$ hMSCs.

Table S2. Differentially expressed proteins (DEPs) between *EIF4EBP1*$^{-/-}$ and *EIF4EBP1*$^{+/+}$ hMSCs.

Table S3. Sequence information of sgRNAs and primers used for gene editing, plasmid construction and qPCR analysis.

Figure S1

A

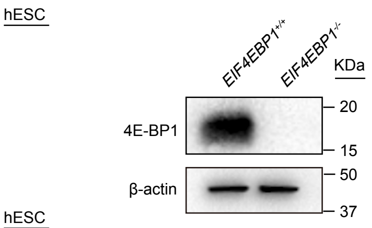

B

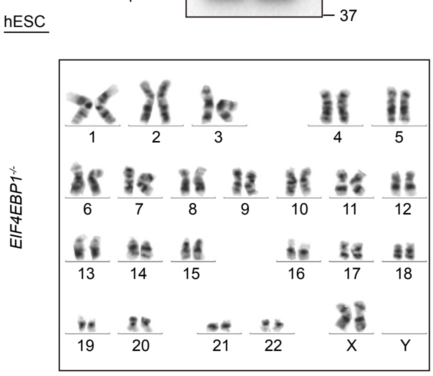

C

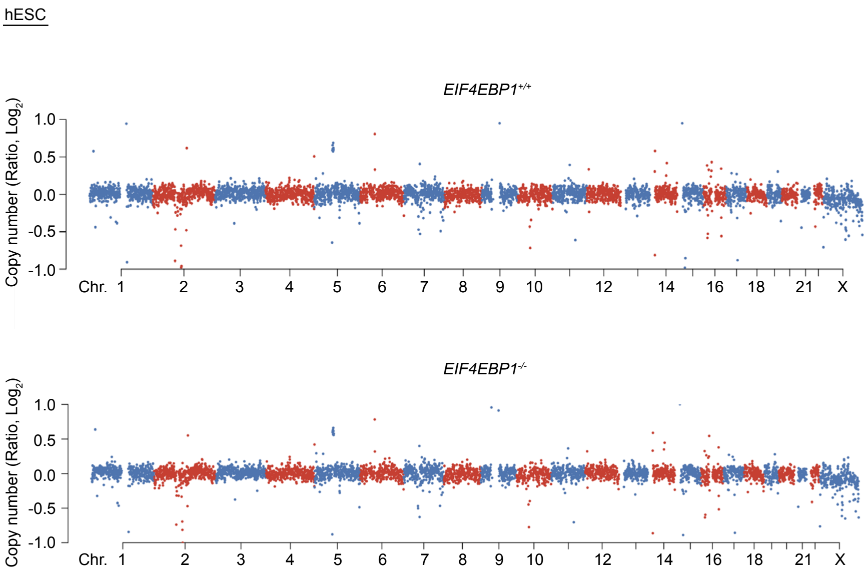

D

hESC

Potential off-target (OT) sites	Predicted off-target scores	Sequences (5'-3')	Locus	Off-target (YES or NO)
On-target	—	GCACCACCCGGCGAGTGGCGGGG	Chr. 8, 38030640 to 38030618	—
OT-1	Score 0.9	ACTCCAGCCAGCGAGTGGCGGGG	Chr. 12, 63844537 to 63844515	NO
OT-2	Score 0.7	GCTCCACCTGGCCAGTGGCGGAG	Chr. 22, 48299225 to 48299247	NO
OT-3	Score 0.5	GGACAACCCAGCGAGTGGTGGAG	Chr. 22, 45061352 to 45061330	NO
OT-4	Score 0.5	GCACCACCCAGTGAGTGGCAGGG	Chr. 14, 21419686 to 21419664	NO
OT-5	Score 0.5	GCTTCAGCCGGTGAGTGGCGTGG	Chr. 11, 64241525 to 64241547	NO
OT-6	Score 0.5	GTGCCAGCCGGGGAGTGGCGAGG	Chr. 7, 1387310 to 1387332	NO

E

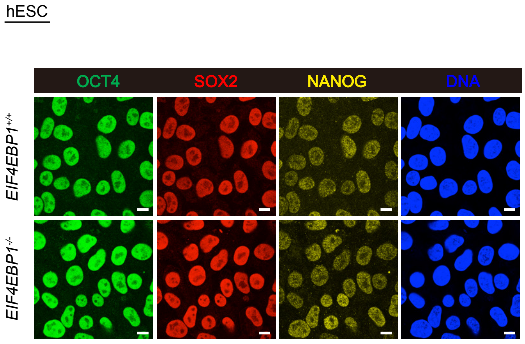

F

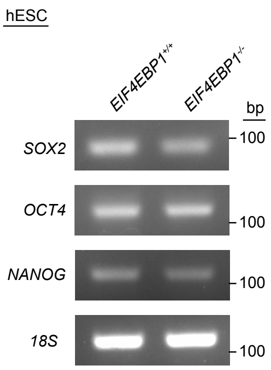

G

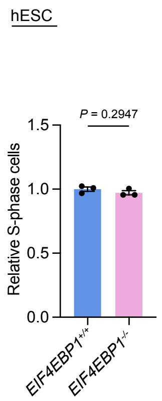

Figure S2

A

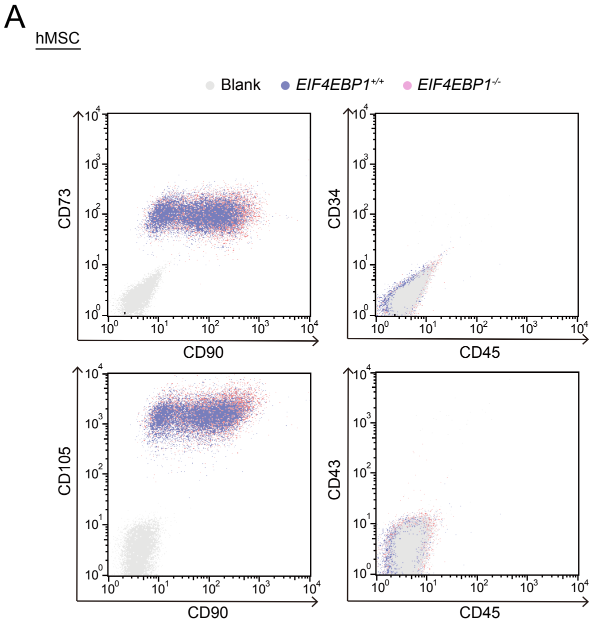

B

hMSC

Surface antigen	Positive cells (%) EIF4EBP1+/+	EIF4EBP1-/-
CD166	99.93 ± 0.06	99.73 ± 0.06
CD29	100.00 ± 0.00	99.83 ± 0.15
CD44	95.50 ± 0.40	95.87 ± 1.55
CD13	96.47 ± 0.31	93.53 ± 0.06
HLA	81.67 ± 0.55	90.53 ± 0.15
CD14	0.02 ± 0.02	0.01 ± 0.01
CD19	0.07 ± 0.04	0.10 ± 0.02
CD164	0.02 ± 0.01	0.04 ± 0.03
PDPN	0.47 ± 0.12	0.39 ± 0.10

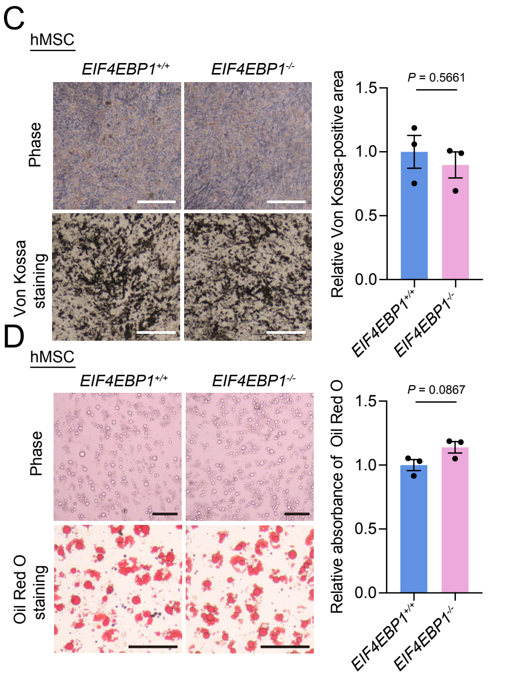

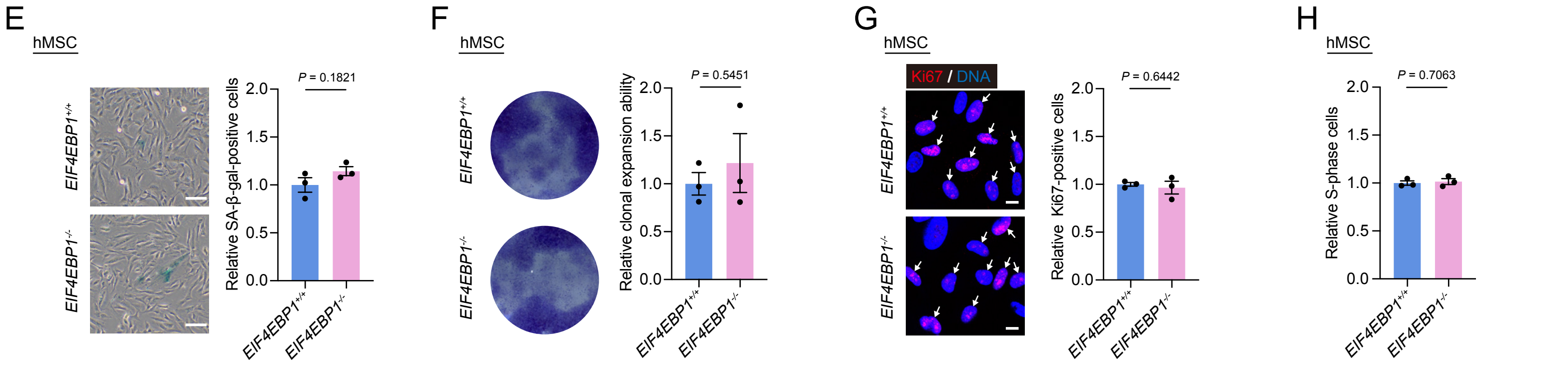

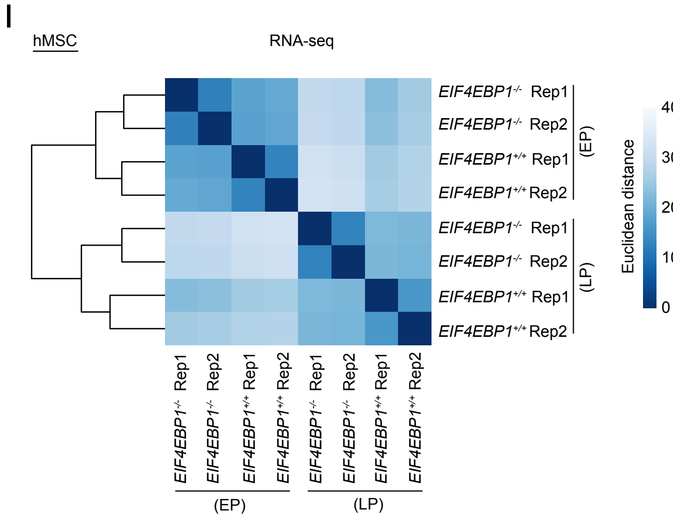

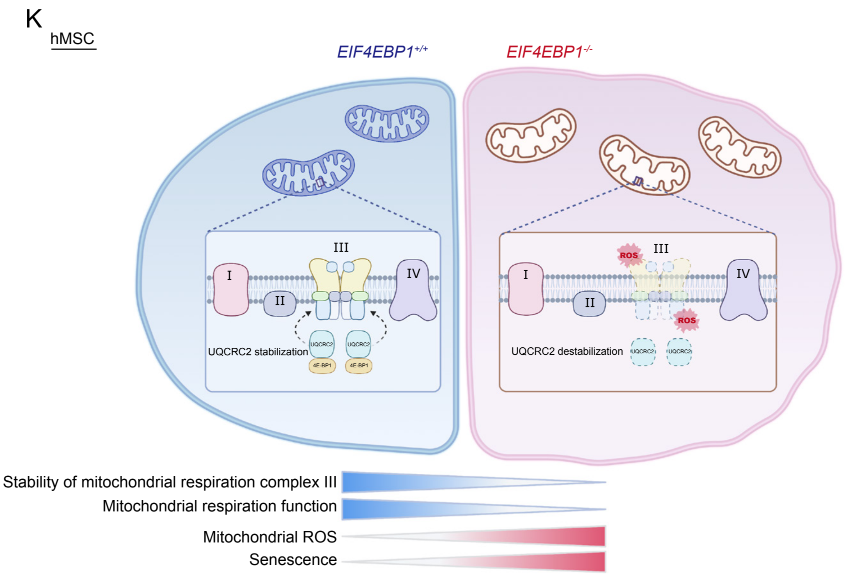

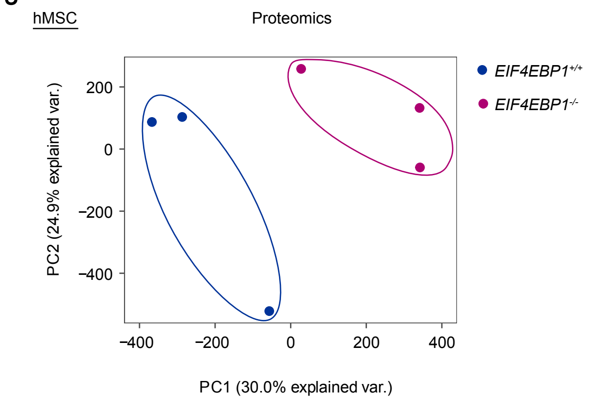

Supplement: pwac037_suppl_Supplementary_Material [file pwac037_suppl_supplementary_material.pdf]
